# Non-Infectious Anterior Uveitis Is Associated with Functional Retinal Changes Demonstrable by Multifocal Electroretinography

**DOI:** 10.3390/jcm15082865

**Published:** 2026-04-09

**Authors:** Danijela Mrazovac Zimak, Nenad Vukojević, Igor Petriček, Tomislav Jukić, Kristina Ana Škreb, Snježana Kaštelan

**Affiliations:** 1 Department of Ophthalmology, University Hospital Centre, 10 000 Zagreb, Croatia; 2 School of Medicine, University of Zagreb, 10 000 Zagreb, Croatia; 3 Faculty of Civil Engineering, University of Zagreb, 10 000 Zagreb, Croatia; 4 Department of Ophthalmology, Clinical Hospital Dubrava, 10 000 Zagreb, Croatia

**Keywords:** acute anterior non-infectious uveitis, multifocal electroretinography, optical coherence tomography, electroretinography, retinal inflammation, macular function

## Abstract

**Introduction**: Although anterior non-infectious uveitis affects the structures of the anterior segment of the eye, (inflammatory) disruption of the hemato–ocular barrier may lead to changes in the structures of the posterior segment of the eye. **Objective**: To evaluate functional retinal changes using multifocal electroretinography (mfERG) and their relationship with structural optical coherence tomography (OCT) parameters in patients with acute anterior non-infectious uveitis (AANU). Methods: This prospective study included 38 eyes of 19 patients diagnosed with unilateral AANU and age-matched healthy fellow eyes as controls. All subjects underwent comprehensive ophthalmological examination, including best-corrected visual acuity (BCVA), spectral-domain OCT, and mfERG testing at baseline, 3 months, and 6 months. mfERG parameters (amplitude and implicit times) were analyzed alongside central field thickness (CFT), macular volume (MV), and average macular thickness (AMT). **Results**: Eyes affected by AANU demonstrated a significant reduction in mfERG response amplitude in the central retinal region compared with control eyes, particularly during the acute phase. Although OCT parameters showed partial structural normalization during follow-up, functional recovery was less pronounced in selected retinal regions. Latency values showed minimal variation over time. These findings indicate a potential dissociation between electrophysiological function and structural morphology during disease resolution. **Conclusions**: Acute anterior uveitis is associated with measurable macular functional impairment detectable by mfERG, even when structural OCT parameters appear relatively stable. These results suggest that inflammatory processes in AAU may extend beyond the anterior segment and transiently affect retinal function. mfERG may therefore serve as a sensitive adjunct tool for detecting and monitoring subclinical macular dysfunction in AANU. **Clinical Relevance**: Functional retinal impairment may persist despite apparent structural recovery in acute anterior uveitis. Incorporating mfERG into clinical evaluation may improve the detection of subtle macular involvement and enhance understanding of disease dynamics beyond conventional imaging findings.

## 1. Introduction

Anterior uveitis (AU) is the most common form of uveitis, accounting for approximately 50–75% of all cases of uveitis [1,2,3,4]. It is an inflammation that affects the anterior part of the middle eye layer, iris, and ciliary body, but may also involve the cornea, anterior chamber (AC), and anterior vitreous. Clinical presentation ranges from a “quiet, white eye” with minimal inflammatory reaction to a very red, painful, eye with moderate-to-severe inflammation [5]. AU can be infectious or noninfectious in etiology. The latter occurs as an isolated process, a post-traumatic process or, often, as part of systemic conditions, such as systemic diseases related to the HLA system, sarcoidosis, multiple sclerosis, psoriatic arthritis, inflammatory bowel disease (IBD), malignancy, etc. [6,7,8,9]. Studies have shown that AU is more associated with genetic factors than with environmental factors [10]. Uveitis in systemic diseases is one of the most common manifestations of the disease outside the primary inflammatory process [11,12,13]. Approximately 20–50% of AU cases are idiopathic anterior uveitis [14,15,16].

More than 90% of uveitis cases’ first appearance occur in patients older than 20 years and subsequent uveitis recurrence in most patients occurs in middle age, at the peak of working life [17,18]. This makes uveitis a major ophthalmic disease with an important socioeconomic impact. Although the prevalence of uveitis is not high compared to other diseases, their complications and impact on society are comparable to the impact of better-known and more common diseases, such as diabetes mellitus. The estimated health care costs for uveitis patients in the United States are $242.6 million per year, while the costs of care for patients with diabetes mellitus are $242.7 million per year [19,20]. Since most uveitis forms and their possible complications can be treated successfully in a timely manner, further research is necessary for both the patient and the entire society.

Previous studies have observed an association between AANU and changes in the posterior eye segment as evidenced by OCT [7,21,22,23,24,25]. The conclusion of all these studies is that structural diagnostics of the posterior eye segment is important in the detection of (sub)clinical structural changes in the retina and choroid in all types of AU.

Only a few studies have examined the role of electrophysiological methods in the diagnosis and monitoring of uveitis [26,27,28,29,30,31,32,33,34,35,36]. Brouwer et al. found that pathological changes in ERG in pediatric non-anterior uveitis occur even when the visual acuity (VA) of the affected eye is normal [27]. In uveitis with unexplained visual loss without pathological changes in retinal structure, ERG changes have been demonstrated, and it has also been established that pathologically altered ERG is associated with the severity of inflammation in non-infectious uveitis of various localizations [29,34]. It was shown that prolonged implicit b-wave time on ERG often occurs at the very beginning of uveitis, which later persists, and that eyes with still active uveitis have worse control ERG results [28]. Georgiadou et al. investigated the correlation between anatomical and functional changes before and after uveitis macular edema (UME) therapy in uveitis and obtained a negative correlation between foveolar thickness and zone 1 mfERG amplitude, thus showing that the impairment of visual function is directly correlated with the degree of macular edema (ME) [37]. The question remains as to what extent visual function has changed in patients with AU who do not yet have visible changes in the posterior eye segment, and whether the extent of subsequent retinal structural changes corresponds to the degree of functional visual impairment.

## 2. Materials and Methods

This prospective study was conducted at the Department of Ophthalmology University Hospital Center Zagreb, during a period of two years. Painless, non-invasive, and harmless methods were used. Adult patients with acute unilateral non-infectious anterior uveitis were included. The first group consisted of 19 eyes with acute anterior non-infectious uveitis, and the second group consisted of the accompanying, healthy eye of the same subject, a total of 38 eyes in the two groups of subjects who met the criteria listed below.

Inclusion criteria for subjects: acutely occurring AANU—first group; structurally and functionally healthy eye of the same subject—second group; subjects aged 18+ (adult subjects)—both groups. Exclusion criteria: complications of the underlying disease that affect the possibility of obtaining clear and comparable test results: cataract that significantly reduces the patient’s VA and visualization of the posterior eye segment (LOCS III system: NO III–NC III and higher, P IV–V), severe posterior synechiae in 180° of the angle, and evidence of an infectious agent. Exclusion criteria for subjects: AU of infectious etiology, uveitis of other localizations (according to the Standardization of uveitis nomenclature—SUN classification), previously diagnosed ophthalmological diseases or conditions with proven changes in the structure or function of the eye (severe amblyopia, glaucoma, advanced cataract, posterior segment diseases, data on inflammatory, traumatic injuries, previous eye surgeries), chronic systemic diseases with proven existing advanced changes in ophthalmological status (diabetes, neurodegenerative diseases), use of medications that could cause retinal changes (e.g., chloroquine, hydroxychloroquine, sildenafil, etc.), refractive error greater than 6 diopters, VA < 0.2 (Snellen chart).

Each examination began with taking a detailed history followed by complete ophthalmological examination. Patients were referred for systemic check-up of uveitis to rule out infectious cause of the disease. Ophthalmological examination consisted of VA determination (Snellen converted to logMAR values for the purposes of statistical analysis), biomicroscopic examination of the anterior eye segment, measurement of intraocular pressure (IOP) by Goldmann applanation tonometry, and indirect ophthalmoscopy. All subjects underwent macular imaging with OCT in mydriasis and mfERG in mydriasis. All subjects were examined by a single examiner on the same Zeiss SL 130 system biomicroscope (serial number 1209911, manufactured by Carl Zeiss, Jena, Germany), OCT images were recorded on the Optopol SOCT Copernicus HR device (serial number 151266/M _CNFF345018, manufactured by Optopol Technology, Zawiercie, Poland), and electroretinography was recorded on the Roland-Consult RETI-port/scan 21 device (serial number 04-99-13031, manufactured by Roland Consult, Brandenburg an der Havel, Germany) according to the ISCEV protocol, using Hawlina–Konec electrodes (manufactured by HK Med, Ljubljana, Slovenia) [38].

All the above was done during the first cycle of monitoring patients with unilateral AANU and at follow-up examinations after three and six months. Given the nature of the condition itself, patients were monitored more frequently, as part of regular work-up, treatment, and monitoring of the acute state of the disease, but three examinations were planned and considered for statistical analysis when diagnostic imaging methods were applied at previously defined time intervals of follow-up examinations.

Statistical processing and data analysis were performed with the program STATISTICA 6.1, StatSoft Inc., Tulsa, OK, USA. The program module Power Analysis-Sample Size Calculation was used to determine the required sample size. The sample size was determined with the standard assumptions for statistical testing that the statistical testing will be carried out at a significance level of 5% (Alpha = 0.05), and that the power of the test is 90% (Power Goal = 0.90), with the assumption for testing the statistical difference within the same group of subjects between three separate examinations in time intervals and between all groups at the same test time that a standardized effect (difference between arithmetic means expressed in standard deviation) greater than 0.6 will be considered statistically significant. With these assumptions, the required sample size was 15 examinees.

The first group consisted of the affected eye in a patient with AANU, and the second, control, group consisted of the healthy eye of the same patient in which there were no signs of uveitis at the time of examination and no anamnestic data on previous uveitis episodes. Statistical testing was performed at a statistical significance level of 5%, confidence level of 95%. General data on the patients were described by descriptive statistics (numerical data) and frequency tables (descriptive data). For comparison between the affected and control eyes in relation to the measured values of OCT and mfERG by examination, analysis of variance for repeated measures (ANOVA) and Fisher’s LSD test within the analysis of variance were used.

## 3. Results

### 3.1. Demographic and Clinical Characteristics of Patients

This prospective study initially included 31 subjects, but five subjects were diagnosed with an infectious cause of AU, and seven subjects were excluded from the statistical analysis because they did not attend regular scheduled follow-up examinations with diagnostic imaging. Data from 19 subjects were collected and analyzed. Demographic and clinical details are presented in Table 1.

Regarding the presence of systemic diagnosis related to immune conditions, four subjects (21.1%) had no previously diagnosed systemic disease, seven subjects (36.84%) had HLA-B27 positive ankylosing spondylitis, two subjects (10.53%) had IBD, three subjects (15.8%) rheumatic disease, and one had autoimmune thyroiditis (5.3%). HLA typing (B and DR loci) was performed on all subjects.

At the first examination, the BCVA of the affected eye had statistically significantly higher logMAR values than at examinations after three and six months (*p* < 0.001) and compared to the control eye (*p* < 0.001).

### 3.2. Optical Coherence Tomography Results

Detailed OCT values are presented in Table 2. Analysis of variance for repeated measures and Fisher’s LSD test within the analysis of variance show that the central field thickness (CFT), macular volume (MV), and average macular thickness (AMT) of the affected eye are statistically significantly higher than those in the control eye at first examination (*p* < 0.05, *p* < 0.001 and *p* < 0.001). Also, MV and AMT of the affected eye are statistically significantly higher at first examination than at the three-month (*p* = 0.035, *p* = 0.047) and six-month examination (*p* = 0.022 and *p* = 0.021).

**Figure 1:**
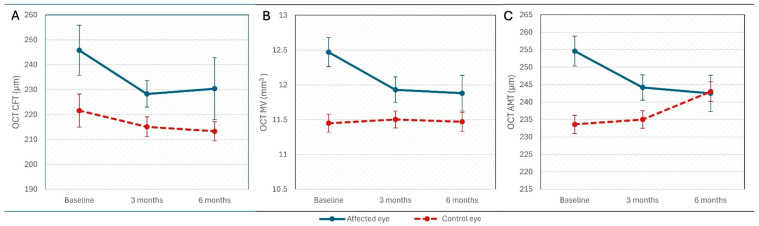
Longitudinal OCT Structural Changes. (**A**) Central field thickness (CFT); (**B**) Macular volume (MV); (**C**) Average macular thickness (AMT). Values represent mean value ± SD vs. control eye or baseline (ANOVA with post hoc LSD test).

Statistical differences are shown in Figure 1.

### 3.3. Multifocal ERG Results

All values of mfERG recording results by rings and quadrants are shown in Table A1 in the Appendix A.

#### 3.3.1. Amplitudes

**Figure 2:**
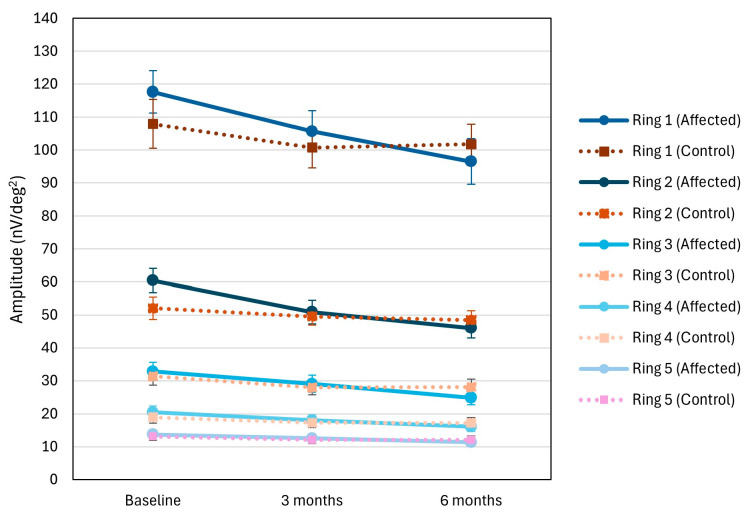
mfERG Amplitudes (Rings 1–5). Mean response amplitudes (nV/deg^2^) across mfERG rings 1–5 in affected (solid line) and control eyes (dashed line) at baseline, 3 months, and 6 months. Significant increase in amplitude in affected eyes at baseline with progressive decline over time. Values are mean value ± SD.

The amplitude of mfERG waves in all rings 1–5 was higher in the affected eye compared to the control eye at the first examination, especially pronounced in the more peripheral rings 3–5 (ring 3 *p* = 0.003; ring 4 *p* = 0.001; ring 5 *p* = 0.005). In all rings of the affected eye during follow-up, there was a significant decrease in amplitude, approaching the values of the control eye (ring 2 *p* = 0.039 and *p* = 0.002; ring 3 *p* = 0.013 and *p* = 0.001; ring 4 *p* = 0.003 and *p* = 0.001; ring 5 *p* = 0.007 and *p* = 0.001) (Figure 2).

**Figure 3:**
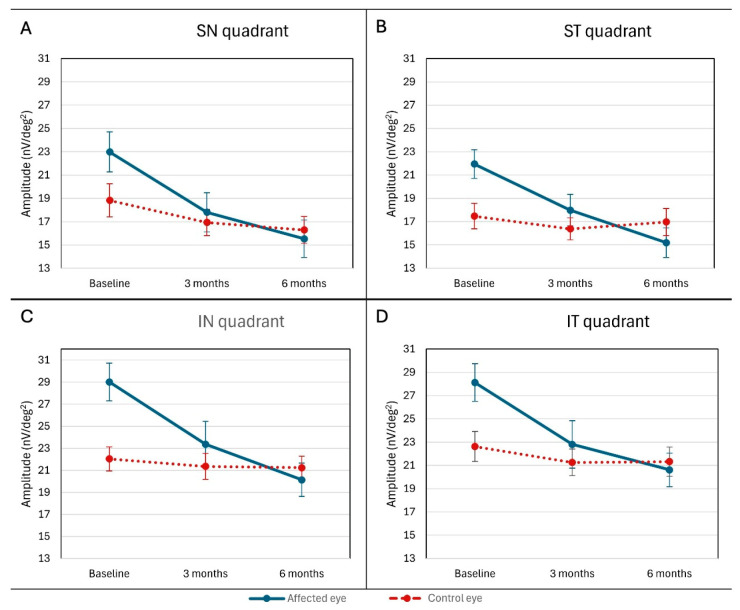
(**A**–**D**) mfERG Amplitudes (Quadrants). Mean response amplitudes (nV/deg^2^) across mfERG quadrants (ST: superotemporal; IT: inferotemporal; IN: inferonasal; SN: superonasal) in affected (solid line) and control eyes (dashed line). Higher amplitudes in affected eyes at baseline (*p* < 0.05 across quadrants), with significant decline after 6 months. Values are mean value ± SD.

The amplitude of mfERG wave in all quadrants (ST, IT, IN, SN) was higher in the affected eye at the first examination than in the control eye (ST *p* = 0.009; IT *p* = 0.011; IN *p* = 0.001; SN *p* = 0.048), with a statistically significant decrease in amplitudes in the affected eye during follow-up and a gradual equalization with the values of the control eye (Figure 3).

#### 3.3.2. Latencies

**Figure 4:**
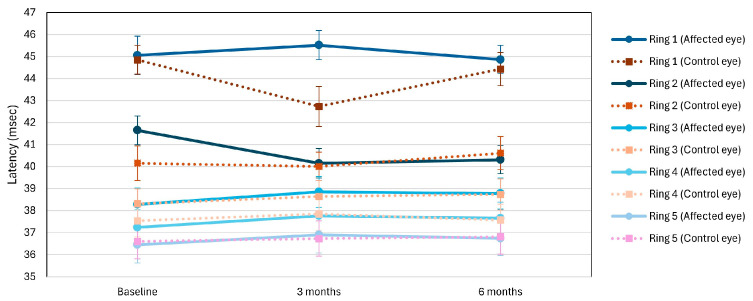
mfERG Latency (Rings 1–5). Mean latency times (ms) across mfERG rings 1–5 in affected (solid line) and control eyes (dashed line). Prolonged latencies in affected eyes at baseline/selective rings. Values are mean value ± SD.

The latency of the wave in ring 1 mfERG in the affected eye was statistically significantly longer than the control eye after three months (*p* = 0.011), while in rings 3 and 4, borderline significant, or significantly longer, latencies were observed in the affected eye at the first examination (ring 3 *p* = 0.051; ring 4 *p* = 0.044), and in rings 2 and 5 there was no significant difference in latencies between the affected and control eyes throughout the observed examinations (Figure 4).

**Figure 5:**
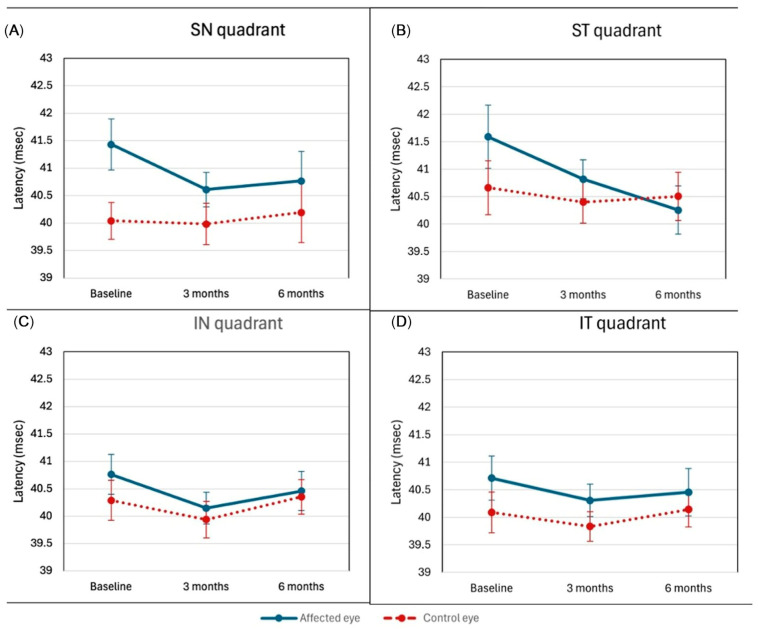
(**A**–**D**) mfERG Latency (Quadrants). Mean latency times (ms) across mfERG quadrants (ST: superotemporal; IT: inferotemporal; IN: inferonasal; SN: superonasal) in affected (solid line) and control eyes (dashed line). Selective prolongation in ST and SN at baseline. Values are mean value ± SD.

The latency of the wave in the ST quadrant was statistically significantly longer in the affected eye at the first examination compared to the control examinations after six months (*p* = 0.040). In the SN quadrant, the latency in the affected eye was significantly longer than in the control eye at the first examination (*p* = 0.028), while in the IT and IN quadrants, no significant difference in latency was found between the affected and control eyes across all three examinations (Figure 5).

#### 3.3.3. Summary of the mfERG Results

In summary, key mfERG findings include: (1) transient amplitude elevation in affected eyes at baseline (all rings/quadrants, *p* < 0.05), followed by significant decline or normalization by 6 months; (2) selective central ring (1–2) changes with incomplete recovery after 6 months; (3) prolonged latencies in acute phase (e.g., ring 1 at 3 months, *p* = 0.011; ST quadrant, *p* = 0.040), resolving variably. These indicate centrally predominant, transient dysfunction dissociating from structural OCT recovery.

### 3.4. Correlations—Clinical and Medical Examinations

#### 3.4.1. Correlations Between Visual Acuity and Degree of Inflammation Versus mfERG Findings

Correlations between BCVA expressed in logMAR values of the affected eye and ERG findings showed that there is a statistically significant association in the parameters shown in Table 3. However, although correlation between the degree of inflammation in the anterior eye segment and the mfERG findings shows that there is a positive or negative correlation, depending on the observed parameters, there was no statistically significant association (*p* > 0.05). Details are shown in Table 4.

#### 3.4.2. Correlations Between OCT and mfERG Findings

Correlation between OCT findings (MV and AMT) and mfERG of the affected eye showed that there was a statistically significant association in certain parameters examined, which are presented in detail in Table 5. Other mfERG parameters did not show a statistically significant correlation with OCT findings. The correlation between CFT and mfERG findings did not show a statistically significant difference in the parameters examined, except for the ST quadrant latency, where it showed a borderline statistically significant association (correlation coefficient 0.453, *p* = 0.0515).

## 4. Discussion

With the introduction of electrophysiological diagnostic tests into regular ophthalmological practice, functional changes in vision related to changes in the posterior eye segment were observed, which in scope do not necessarily follow changes in its structures [39]. Thus, Pescosolido et al. examined the role of electrophysiological tests in the diagnosis and monitoring of diabetic retinopathy (DR) and concluded that, before structural changes can be clinically diagnosed, there are already functional changes in the retina that can be proven by electroretinography and recommended that such tests should be included in the regular monitoring of DR [40]. On the other hand, Yamamoto et al. showed that mfERG findings are a good objective indicator of macular function in patients with diabetic ME and that they are directly related to morphological changes in the macula [41]. As early as 1987, Bloch-Michel et al. proposed the use of this method in the treatment and monitoring of uveitis through the IUSG [42]. Although there are not many such studies, a few scientists have thus far investigated the role of electrophysiological methods in the diagnosis and monitoring of inflammatory changes in the choroid and retina [1,26,31,35]. In their study published in 2019, Brouwer et al., in which they analyzed ERG changes in pediatric non-anterior uveitis, concluded that ERG results were changed in more than half of the subjects [27]. In uveitis with unexplained vision loss without pathological changes in retinal structure, changes in ERG have been demonstrated, and it has also been found that pathologically altered ERG is associated with the severity of inflammation in non-infectious uveitis of various localizations [29,34]. The demonstrated changes in ERG function indicate that such retinal damage may occur early in the process of inflammatory changes. In a minority of cases, the resolution of the inflammatory process may result in recovery of retinal function, thus emphasizing the importance of early and adequate treatment of uveitis [28]. Georgiadou et al. investigated the correlation between anatomical and functional changes before and after UME therapy in uveitis and obtained a negative correlation between foveolar thickness and zone 1 mfERG amplitude, thus showing that the impairment of visual function is directly correlated with the degree of ME. Also, the results of their study showed that pre-treatment mfERG values were associated with CFT and VA, but that the decrease in CFT values after treatment of ME was not accompanied by an improvement in mfERG values and VA. Thus, they showed that CFT alone after UME therapy is not a good predictor of improvement in macular function [37].

In this study, we observed a particularly interesting finding: in the acute phase of AANU, the amplitudes of all mfERG waves in the affected eyes were higher than those of the fellow control eyes recorded at the same time point, a phenomenon not previously described in the literature. We hypothesize that this transient increase may reflect globally enhanced ocular circulation and retinal sensitivity during acute inflammation, consistent with reported photophobia, but this remains speculative and warrants confirmation in future studies with vascular imaging correlates.

The mfERG results also demonstrate clear functional retinal changes with predominant central involvement. The marked reduction in amplitudes in Rings 1 and 2 of the affected eyes, particularly the progressive decline from baseline to the 6-month visit, suggests early macular dysfunction typical of uveitis-related inflammatory disturbance. The partial recovery of these values at 6 months, approaching those of the control eyes, indicates functional improvement under treatment, whereas the relatively preserved responses in Rings 4 and 5 support a central-to-peripheral gradient of retinal involvement.

Furthermore, mfERG analysis in our cohort shows that the latencies of all analyzed segments are prolonged in the acute phase, most often to a statistically significant extent, consistent with an acute inflammatory state and transient disturbance of signal transmission between photoreceptors due to intercellular edema. Over time, amplitudes decrease across rings and quadrants, indicating that functional retinal changes have occurred and may persist beyond the initial inflammatory episode. This raises the question of whether a sustained reduction in mfERG amplitudes over the follow-up period should be considered an early marker of functional involvement of the posterior segment, even when no structural abnormalities are detectable on conventional imaging, and whether it may help predict the timing and extent of permanent visual function loss [1].

Finally, the spatially resolved nature of mfERG allows topographic mapping of retinal function, so the location of dysfunction may serve as an indicator of its potential impact on future vision. For example, localized dysfunction in regions prone to subsequent edema near the fovea is likely to be clinically more relevant than similar changes confined to the peripheral retina [43,44].

We did not present the ring ratio in the mfERG results because this ratio does not have generally accepted reference intervals. It has been shown that values in the general population, especially the ring ratio 1, vary widely and such a comparison lacks statistical power in data analysis [45].

The findings of Brouwer et al. indicate that ERG abnormalities occur in noninfectious uveitis of various locations, including AAU in which there is no evidence of inflammation or changes in the posterior eye segment. ERG abnormalities appear to be associated with both current inflammatory changes in the posterior eye segment and previous inflammation. Therefore, such abnormalities are present even when there are no longer any current signs of inflammation in the anterior eye segment [28,29]. This leads to the conclusion that it is necessary to treat inflammation promptly because chronic inflammation results in worse functional visual outcomes [46].

In our study, we showed that mfERG wave latencies are also affected by inflammation and that the changes persist even after six months. Although the degree of correlation was clearly present, there was no statistically significant association. We believe that this difference would have been significant if the sample size had been larger, as the results correspond to observed changes from a clinical perspective.

Prolongation of latency is also related to age, and it is possible that the patient’s age could serve as a predictor of changes in ERG latencies in uveitis patients. From an electrophysiological point of view, prolongation of latency is an indicator of altered transmission of the electrical signal from photoreceptors to bipolar and Müller cells. One of the reasons for altered transmission is the damage of synapses. In most eye diseases, latency prolongation is related to decrease in amplitude; however, Brouwer et al. believe that only latency is often affected in uveitis. Researchers believe that ERG is very useful in detecting early signs of retinal damage, before visible anatomical changes can be proven by OCT, and that ERG could be useful in the evaluation of such patients [28]. Through our research, we have shown that there are several changed variables and that it is important to look at them in the context of clinical changes in order to correctly interpret these data.

There is a lot of research in the field of uveitis that investigates changes in retinal structures affected by uveitis, as well as many studies related to monitoring the treatment effects of uveitis complications, but there are no available studies related to determining the correlation of macular structural and functional changes associated with AU [1,32,37,43,47].

In our research, we compared the data of macular structures using OCT, which include CFT, MV, and AMT with data of macular function using mfERG, which also include amplitudes and latencies of rings and quadrants in macula. Results of mfERG correlations with MV and AMT indicate that in the active phase of inflammation there is a positive correlation between the latencies of individual mfERG segments, which indicates a dysfunction of transmission in the inner layers of the retina from photoreceptors to bipolar cells. Such dysfunction in the inflammation phase is explained by the disturbed structural relations between neuronal cells and by the weakening of the signal in the communication sequence that occurs due to the disruption of the haemato–ocular barrier. Although the results of positive correlation of CFT with mfERG parameters did not show a statistically significant value, i.e., only in one parameter a marginal statistically significant value, we believe that it exists due to the nature of the process. It is clinically important and we believe that such a difference would also prove significant when including a larger number of subjects with the same pathology.

The primary limitation of this study is the relatively small sample size. However, considering the incidence of AANU and the proportion of affected patients treated in a tertiary referral center, the number of included subjects was sufficient to allow meaningful statistical analysis. A post hoc power analysis indicated that the study was adequately powered to detect moderate-to-large effect sizes, although it limits detection of subtle differences and generalizability. Nevertheless, the limited sample contributed to wider confidence intervals in certain parameters, which may have reduced the statistical power to detect smaller effect sizes.

An additional limitation relates to the inclusion criteria for visual acuity (VA), which were determined by the technical requirements for reliable mfERG recording. Patients with VA below 0.2 in the acute phase were therefore excluded. While this approach ensured methodological consistency and signal quality, it may have limited the representation of more severe disease presentations. Inclusion of such patients might have resulted in stronger associations between functional and structural parameters, as suggested by previous research examining complications of anterior uveitis.

Future studies with larger cohorts and broader inclusion criteria are essential to confirm the observed trends and to further clarify the relationship between electrophysiological and morphological changes in AANU.

## 5. Conclusions

This study demonstrates that AANU is associated with measurable functional retinal changes detectable by mfERG, even in the absence of pronounced structural alterations on OCT. The observed reduction in central retinal response amplitude suggests that inflammatory processes in AAU may extend beyond the anterior segment and transiently affect macular function. Although structural OCT parameters showed partial recovery over time, functional alterations persisted in certain retinal regions, indicating a potential dissociation between morphological normalization and electrophysiological recovery. These findings support the concept that AAU may have a broader retinal impact than previously assumed. Multifocal ERG may therefore represent a sensitive adjunct tool for monitoring macular function in AAU, particularly in cases where structural imaging appears stable. Multifocal ERG could refine routine management by objectively detecting subclinical macular dysfunction in AANU patients with unexplained visual complaints or disproportionate symptoms despite normal OCT or visual acuity findings. This could enable earlier intensification of therapy (e.g., topical or periocular steroid therapy). We recommend it primarily for selected clinical cases (e.g., persistent symptoms or high-risk systemic disease) and research monitoring, pending cost effectiveness validation and standardized protocols. Further prospective studies with larger cohorts are essential to confirm these findings and to clarify the long-term functional implications of anterior segment inflammation.

## Figures and Tables

**Table 1:** Demographic and Clinical Characteristics.

Characteristic	Patients/Values	
**Demographic Characteristics**		
Sex	12 women (63.2%), 7 men (36.8%)	
Age	Mean 46.9 years (median 45), range 21–69 years	
Age at first onset of uveitis	Mean 43.9 years (median 43)	
Duration from the first onset	Mean 2.9 years, range 0–15 years	
Uveitis type	First episode: 11 patients (57.9%) Recurrent: 8 patients (42.1%)	
Systemic diagnosis	Known: 15 patients (78.9%) Unknown: 4 patients (21.1%)	
**HLA Typing—Main Findings**		
HLA B27	11 patients (57.9%)	
HLA B18	4 patients (21.1%)	
HLA A2/HLA DR7	3 patients (15.8%)	
HLA-A1/A32/B13/B44/DR4/DR13/DR16	2 patients (10.5%)	
Other HLA types (A3/A23/A24 /A25/A29/A30/A33/A68/B7/B14/B17 /B35/B40/B55/B57/B60/DR8/ DR11/DR14/DR15/DR17)	1 patient (5.3%)	
**Clinical Characteristics**		
	**Affected Eye**	**Control Eye**
**BCVA (logMAR)**		
1st exam	0.163 ± 0.167	0.005 ± 0.023
3-month follow-up	0.037 ± 0.083	0.005 ± 0.023
6-month follow-up	0.037 ± 0.083	0.005 ± 0.023
**Inflammation (SUN scale)—**1st exam		
0+	0 (0%)	0 (0%)
0.5+	2 (10.53%)	0 (0%)
1+	12 (63.16%)	0 (0%)
2+	3 (15.79%)	0 (0%)
3+	2 (10.53%)	0 (0%)
**Inflammation (SUN scale) 2nd & 3rd exam**		
0+	19 (100%)	19 (100%)

HLA—human leukocyte antigen; BCVA—best-corrected visual acuity; logMAR—logarithm of the Minimum Angle of Resolution; SUN—Standardization of Uveitis Nomenclature.

**Table 2:** Longitudinal OCT Structural Parameters.

Parameter	Time Point	Affected Eye (Mean ± SD)	Control Eye (Mean ± SD)	*p*-Value
CFT (µm)	Baseline	245.8 ± 43.9	221.6 ± 29.0	*p* < 0.05
	3 months	228.3 ± 23.1	215.1 ± 17.2	*p* > 0.05
	6 months	230.4 ± 54.2	213.3 ± 16.7	*p* > 0.05
MV (mm^3^)	Baseline	12.47 ± 0.91	11.45 ± 0.56	*p* < 0.001
	3 months	11.93 ± 0.80	11.50 ± 0.53	*p* = 0.035
	6 months	11.88 ± 1.12	11.47 ± 0.60	*p* = 0.022
AMT (µm)	Baseline	254.6 ± 18.7	233.6 ± 11.5	*p* < 0.001
	3 months	244.2 ± 15.8	235.0 ± 10.9	*p* > 0.05
	6 months	242.5 ± 22.7	243.0 ± 12.2	*p* > 0.05

CFT—central field thickness; MV—macular volume; AMT—average macular thickness; SD—standard deviation.

**Table 3:** Correlation Between BCVA and mfERG Parameters *.

mfERG Parameter	BCVA cc	*p*-Value
Ring 1 latency (ms) *	0.2531	0.007
Ring 2 latency (ms)	0.2343	0.012
Ring 4 amplitude (nV/deg^2^)	0.2604	0.005
Ring 4 latency (ms)	0.2593	0.005
Ring 5 amplitude (nV/deg^2^)	0.2148	0.022
Ring 5 latency (ms)	0.2094	0.025
ST quadrant amplitude (nV/deg^2^)	0.2469	0.008
ST quadrant latency (ms)	0.2328	0.013
IN quadrant latency (ms)	0.1890	0.044
SN quadrant latency (ms)	0.2217	0.018

mfERG—multifocal electroretinography; BCVA—best-corrected visual acuity; cc—correlation coefficient; ST—superotemporal; IN—inferonasal; SN—superonasal; * *p* > 0.05.

**Table 4:** Association Between Degree of Inflammation and mfERG Parameters *.

mfERG Parameter	Direction of Correlation
Ring 1–4 amplitude	Positive
SN, IN, IT amplitudes	Positive
Ring 5 amplitude	Negative
ST amplitude	Negative
All quadrant latencies	Negative

mfERG—multifocal electroretinography; ST—superotemporal; IN—inferonasal; SN—superonasal; IT—inferotemporal; * *p* > 0.05.

**Table 5:** Correlations Between OCT Structural Parameters and mfERG Latency Values.

mfERG Parameter (Latency)	OCT Macular Volume (mm^3^)	*p*-Value	OCT Average Macular Thickness (µm)	*p*-Value
Ring 2	0.550	0.015	0.554	0.014
Ring 5	0.480	0.038	0.480	0.038
IT quadrant	0.461	0.047	0.462	0.046
SN quadrant	0.592	0.008	0.595	0.007

mfERG—multifocal electroretinography; OCT—optical coherence tomography; IT—inferotemporal; SN—superonasal.

## Data Availability

The data presented in this study are available on request from the corresponding author. The data are not publicly available due to privacy and ethical restrictions.

## Abbreviations

The following abbreviations are used in this manuscript:

AU	Anterior uveitis
ANU	Anterior non-infectious uveitis
AANU	Acute anterior non-infectious uveitis
IBD	Inflammatory bowel disease
OCT	Optical coherence tomography
ERG	Electroretinography
mfERG	Multifocal electroretinography
ffERG	“Full-field” electroretinography
DR	Diabetic retinopathy
IOP	Intraocular pressure
LOCS	Lens opacity classification system
ST	Superotemporal
IT	Inferotemporal
SN	Superonasal
IN	Inferonasal
MV	Macular volume
CFT	Central field thickness
AMT	Average macular thickness
cc	Correlation coefficient
BCVA	Best-corrected visual acuity
logMAR	Logarithm of the Minimum Angle of Resolution
AC	Anterior chamber
UME	Uveitic macular edema
ME	Macular edema
SUN	Standardization of Uveitis Nomenclature
VA	Visual acuity
ISCEV	International Society for Clinical Electrophysiology of Vision

## Appendix A

**Table A1:** Multifocal electroretinography (mfERG) response amplitude and latency values by retinal ring and quadrants, first examination and follow-up visits after 3 and 6 months. Values presented as mean ± standard deviation (SD). Rings 1–5 represent concentric retinal zones from 0–10° (Ring 1) to outer peripheral regions.

Ring	Exam	Eye	Amplitude (nV/deg^2^)		Latency (msec)	
			Mean	SD	Mean	SD
**IT quadrant**	1st visit	Affected	28.118	7.034	40.711	1.746
		Control	22.621	5.667	40.089	1.619
	3-month control	Affected	22.797	8.850	40.305	1.286
		Control	21.253	4.973	39.832	1.165
	6-month control	Affected	20.612	6.275	40.453	1.886
		Control	21.324	5.496	40.142	1.381
**ST quadrant**	1st visit	Affected	21.942	5.393	41.589	2.514
		Control	17.474	4.779	40.663	2.144
	3-month control	Affected	17.980	5.933	40.816	1.542
		Control	16.377	4.093	40.400	1.691
	6-month control	Affected	15.188	5.577	40.253	1.920
		Control	16.966	5.022	40.505	1.918
**IN quadrant**	1st visit	Affected	29.013	7.412	40.763	1.581
		Control	22.043	4.763	40.289	1.600
	3-month control	Affected	23.370	9.062	40.147	1.268
		Control	21.355	5.165	39.937	1.447
	6-month control	Affected	20.141	6.539	40.458	1.559
		Control	21.240	4.504	40.353	1.374
**SN quadrant**	1st visit	Affected	22.980	7.500	41.432	2.034
		Control	18.823	6.183	40.042	1.463
	3-month control	Affected	17.799	7.369	40.611	1.367
		Control	16.937	4.945	39.984	1.642
	6-month control	Affected	15.533	6.983	40.768	2.349
		Control	16.291	4.948	40.195	2.395
**Ring 1**	1st visit	Affected	117.655	27.863	45.047	3.766
		Control	107.964	32.554	44.842	2.858
	3-month control	Affected	105.683	27.267	45.511	2.899
		Control	100.762	27.102	42.726	3.955
	6-month control	Affected	96.504	30.155	44.858	2.811
		Control	101.786	26.375	44.426	3.300
**Ring 2**	1st visit	Affected	60.406	15.933	41.642	2.850
		Control	52.044	14.987	40.147	3.416
	3-month control	Affected	50.901	15.567	40.142	2.925
		Control	49.557	11.392	40.000	2.810
	6-month control	Affected	46.006	13.161	40.305	2.788
		Control	48.484	12.239	40.605	3.261
**Ring 3**	1st visit	Affected	32.825	12.068	38.269	3.269
		Control	31.328	11.647	38.305	2.938
	3-month control	Affected	29.042	11.486	38.842	3.066
		Control	27.949	9.841	38.631	3.143
	6-month control	Affected	24.823	8.865	38.769	3.093
		Control	28.043	10.261	38.726	3.083
**Ring 4**	1st visit	Affected	20.455	8.342	37.233	3.469
		Control	18.939	7.539	37.531	3.209
	3-month control	Affected	18.033	7.547	37.759	3.274
		Control	17.342	6.894	37.842	3.321
	6-month control	Affected	16.088	6.721	37.647	3.156
		Control	17.256	7.183	37.563	3.147
**Ring 5**	1st visit	Affected	13.692	5.829	36.447	3.628
		Control	13.124	5.452	36.598	3.413
	3-month control	Affected	12.541	5.381	36.891	3.547
		Control	12.089	4.967	36.724	3.482
	6-month control	Affected	11.335	4.835	36.734	3.389
		Control	12.087	5.121	36.812	3.425

nV/deg^2^ = nanovolt per square degree; msec = millisecond; ST—superotemporal; IT—inferotemporal; SN—superonasal; IN—inferonasal.
